# Enhanced polyunsaturated fatty acids production in *Mortierella alpina* by SSF and the enrichment in chicken breasts

**DOI:** 10.3402/fnr.v60.30842

**Published:** 2016-10-14

**Authors:** Shengli Yang, Hui Zhang

**Affiliations:** 1 The College of Pharmaceutical Science, Zhejiang University of Technology, Hangzhou, People’s Republic of China; 2 Physical and Chemical Test Center, Zhejiang Institute of Quality Inspection Science, Hangzhou, China

**Keywords:** PUFA, DDGS, *Mortierella alpina*, soybean meal, solid-state fermentation, chicken breasts, enrichment

## Abstract

**Background:**

Distiller’s dried grains with solubles (DDGS) and soybean meal were used as the substrates for the production of polyunsaturated fatty acids (PUFA) in solid-state fermentation (SSF) by *Mortierella alpine*. These fermented products were fed to laying hens. PUFA enrichment from chicken breasts was studied.

**Methods:**

The maximum productivity of PUFA was achieved under optimized process condition, including 1% w/w yeast extract as additive, an incubation period of 5 days at 12°C, 10% v/w inoculum level, 75% moisture content, and pH 6.0. The hens were then fed with ration containing soybean DDGS, rapeseed oil, soybean oil, and peanut oil. The control group was fed with basal ration.

**Results:**

Under the optimal condition, *M. alpine* produced total fatty acids (TFA) of 182.34 mg/g dry substrate. It has better mycelial growth when soybean meal was added to DDGS (SDDGS). PUFA in fermentation product increased with higher soybean meal content. The addition of 70% soybean meal to DDGS substrate yielded 175.16 mg of TFA, including 2.49 mg eicosapentaenoic acid (EPA) and 5.26 mg docosahexaenoic acid (DHA). The ratios of ω-6/ω-3 found in chicken breasts fat were all lower than that found in control by 36.98, 31.51, 18.15, and 12.63% for SDDGS, rapeseed oil, soybean oil, and peanut oil, respectively.

**Conclusions:**

This study identified an optimized SSF process to maximize PUFA productivity by *M. alpine* as the strain. This PUFA-enriched feed increased the PUFA contents as well as the proportions of ω-6 and ω-3 in chicken breasts and liver.

Fatty acids are the major energy source and reservoir for animals to grow and survive (1–3). However, not all the fatty acids are equal. Dietary saturated fat intake has been associated with increased risk of cardiovascular disease. As substitutes of saturated fatty acids, polyunsaturated fatty acids (PUFA) provide many health benefits (4, 5), for example, a reduction in low-density lipoprotein or bad cholesterol and an increase in high-density lipoproteins or good cholesterol (6). Moreover, PUFA contain essential fatty acids like omega-3 and omega-6, which the body need but are not able to synthesize. Eicosapentaenoic acid (EPA), found in fatty cold-water fish and fish oil supplements along with docosahexaenoic acid (DHA) (7), is one of the several omega-3 fatty acids used by the body. Omega-3 fatty acids help lower the risk of heart disease. Increased intake of EPA has beneficial effects on coronary heart disease, high blood pressure, and inflammatory disorders, such as rheumatoid arthritis (8). Most people in the Western countries do not get enough omega-3 fatty acids in their diet, which is one of the reasons that PUFA production has become a prominent research focus.

Microorganisms have been used in PUFA production in recent years (9–15). Several *Mortierella* strains are reported (16–18). The most common *Mortierella* species are *M. alpine* (19, 20) and *M. isabellina* (21, 22). Among the large amounts of published information on microbial PUFA production, EPA (23–28), arachidonic acid (AA) (29–32), and γ-linolenic acid (GLA) (33, 34) are the most popular targets.

Agricultural coproducts such as soybean meal, distiller’s dried grains with solubles (DDGS), rice bran, and wheat bran are abundantly produced. Both soybean meal and DDGS are good substrates for enzymes to produce oil. Being economically competitive, soybean meal and DDGS were used as the base substrate in the present study to produce PUFA using *M. alpina* by solid-state fermentation (SSF).

Adding lipids in feed has been in place for more than 50 years, since 1953. Lipids improve the energy density of the diet and provide essential fatty acids for poultry. Lipids also improve the palatability and processing properties of feed. Essential fatty acids can inhibit the inflammation, promote brain development, inhibit tumor growth, as well as can reduce the plasma triglyceride and cholesterol levels to inhibit thrombosis and atherosclerosis (35).

Fermentation using agricultural coproducts as a substrate can produce a variety of unsaturated fatty acids. PUFA, especially EPA and DHA deposition in poultry, are very important as we essentially need them for our health. The objectives of this study were to determine the parameters for the growth of the fungi selected and to examine the PUFA changes after fermentation. This study at the current stage is a proof of concept in nature. This PUFA-enriched feed increased the PUFA contents as well as the proportions of ω-6 and ω-3 in chicken breasts and liver.

## Materials and methods

### Microorganisms

*Mortierella alpina* was maintained on potato dextrose (PD) agar slants at 4°C and transferred every 3 months.

### Seed culture and inoculum preparation

The fungi were cultivated on PD agar containing 2.0% agar and incubated at 20°C for 7 days until complete sporulation. The spores from the agar were suspended in 15% glycerol. The suspension was used as inoculums (10^7^ spores/mL).

### Solid-state fermentation

Two miniliter spore suspensions of *M. alpina* were transferred to shake flasks (250 mL) containing 50-mL PD medium. Flasks were shaken at 20°C and 150 rpm for 24 h. Inoculum (10% v/w) was transferred to 40 g (as is) substrate in a 250-mL Erlenmeyer flask. The moisture contents of soybean meal and DDGS were adjusted to 75% and the pH to 6.0 before inoculation. Samples were incubated for 10 days. A water reservoir was placed in the incubator to maintain constant moisture content. All treatments were performed in triplicate.

### Optimization of process parameters

SSF was conducted to optimize various process parameters influencing PUFA production, including incubation temperature (10, 12, 15, 20, and 25°C), incubation time, initial moisture content of the substrate (60, 65, 70, 75, and 80%), inoculation volume (5, 8, 10, 12, 15, and 20%), and initial pH (5, 5.5, 6, 6.5, 7, 7.5, and 8). All treatments were performed in triplicate.

### Effect of carbon source on DDGS SSF

DDGS was supplemented with different carbon sources (1% w/w) such as glucose, maltose, fructose, sucrose, and starch to study their effects on PUFA production. The substrate moisture content was 75% and pH was 6.0. All samples were incubated for 5 days at 20°C and then at 12°C for an additional 5 days. All treatments were performed in triplicate.

### Effect of nitrogen source on DDGS SSF

Given the poor growth of the fungi on DDGS, nitrogen sources (1% w/w), such as ammonium sulfate, urea, peptone, casein, and yeast extract, were added into DDGS to examine their effect on PUFA production. All treatments were performed in triplicate.

### Effect of soybean meal addition on DDGS SSF

The moisture contents of soybean meal and DDGS were adjusted to 75%. Soybean meal was added at various concentrations (0, 30, 40, 50, 60, and 70%, as is) to the DDGS substrate to enhance the PUFA production. The substrate pH was adjusted to 6.0, and fermentation was conducted for 5 days at 20°C and then at 12°C for an additional 5 days. All treatments were performed in triplicate.

### Test animals and feeds

Sixty healthy 52-week-old laying hens were selected and divided into four treatment groups based on weights. Each treatment was repeated five times, with four laying hens for each repetition. Single-factor completely randomized design was adopted in the test. Test Groups 1, 2, 3, and 4 were fed with a recipe containing soybean DDGS (SDDGS), 3% rapeseed oil, 3% soybean oil, and 3% peanut oil, respectively. The control group was fed with base recipe. The test period was 45 days.

### Sample collection and analytical methods

During the study period, eggs were collected and counted; the weight of the eggs and the ration consumption were determined. This study was approved by the Animal Ethics Committee in Zhejiang University (*Institutional Animal Welfare and Ethics Committee* in Zhejiang University, China). At the end of the test period, the laying hens were sacrificed by cervical dislocation, and their liver and breast were obtained for later use.

### PUFA analysis

Fermented flour (1 g) was soaked in 30 mL of chloroform/methanol (2:1, v/v) supplemented with 1.5 mL sulfuric acid. Mixture was refluxed for 1 h and reaction was stopped by adding 5 mL deionized water. The lipid phase was then dried. Fatty acid methyl esters were extracted using hexanes and analyzed using capillary gas chromatography (GC; Hewlett-Packard model 5890 series II) equipped with flame ionization detector. The conditions used for GC were as follows: injection temperature, 230°C; detector temperature, 230°C, oven temperature was programmed from 130 to 220°C with a heating rate of 10°C/min. The column used was a supelco sp-2330 (Bellefonte, PA) capillary column, 15 m (length)×0.25 nm (i.d.)×0.2 µm (film thickness).

### Statistical analyses

All treatments were repeated as described under each experiment. ANOVA was performed using SAS and the least significant difference mean comparison was used to compare treatment mean differences at *p=*5%.

## Results

### PUFA accumulation by SSF with M. alpina using DDGS and soybean meal as substrates

As shown in Table 1, each gram of soybean meal substrate yielded 58.75 mg of total fatty acids (TFA), including 0.91 mg EPA, 2.10 mg DHA, 23.65 mg linoleic acid (LA), and 2.29 mg α-linolenic acid (ALA) after a 10-day incubation. As shown in Table 2, each gram of DDGS yielded only 0.42 mg EPA and 0.95 mg DHA, in comparison. This also indicates that soybean meal may be a better nitrogen source for *M. alpina* than DDGS. Therefore, the addition of soybean meal to DDGS may help improve SSF performance.

**Table 1:** Polyunsaturated fatty acids accumulation from *M. alpina* for SSF on soybean meal after 10-day incubation

PUFA name	PUFA formation (original), mg/g dry substrate	PUFA formation (fermented), mg/g dry substrate
16:0	1.33±0.17	9.96±0.82*
18:0	0.50±0.04	2.63±0.23*
18:1	1.63±0.24	12.91±1.18*
18:2	2.01±0.21	23.65±2.84*
18:3	0.27±0.02	2.29±0.331*
20:5	0.00	0.91±0.04*
22:6	0.00	2.10±0.22*
Total	6.08	58.75

PUFA, polyunsaturated fatty acids; SSF, solid-state fermentation.

* *p*<00.05 compared with unfermented group.

**Table 2:** Polyunsaturated fatty acids accumulation from *M. alpina* for SSF on DDGS after 10-day incubation

PUFA name	PUFA formation (original), mg/g dry substrate	PUFA formation (fermented), mg/g dry substrate
16:0	14.81±1.08	15.48±1.40
18:0	2.00±0.19	2.32±0.18*
18:1	25.70±2.32	25.50±2.06
18:2	57.46±4.88	56.42±5.71
18:3	2.11±0.24	2.43±0.28
20:5	0.00	0.42±0.03*
22:6	0.00	0.95±0.08*
Total	105.16	108.32

DDGS, distiller’s dried grains with solubles; PUFA, polyunsaturated fatty acids; SSF, solid-state fermentation.

* *p*<00.05 compared with unfermented group.

### Effect of incubation temperature and time on PUFA content of DDGS SSF

The maximum PUFA yield was obtained at 12°C; however, the microbial growth was better at 20°C. The strains hardly synthesized EPA and DHA when the temperature exceeded 20°C. The detailed results are presented in Table 3. A decrease in the PUFA yield was observed when the incubation temperature was higher or lower than 12°C, indicating that 12°C is more advantageous for PUFA production by *M. alpina*. Although a higher temperature was beneficial to mycelial growth, some adverse effects on the metabolic activities were nevertheless observed. Therefore, we designed a two-phase incubation process with programmed temperatures, that is, 5-day preincubation at 20°C and an additional incubation period (2, 3, 4, 5, 6, and 7 days) at 12°C. The maximum PUFA yield was obtained after 5-day preincubation at 20°C followed by another 5-day incubation at 12°C (Table 4).

**Table 3:** Effect of incubation temperature on PUFA from *M. alpina* for SSF on DDGS

	PUFA (mg/g dry substrate)							
	
Temp. (°C)	16:0	18:0	18:1	18:2	18:3	20:5	22:6	Total
10	15.35±1.32^b^	2.17±0.28^c^	24.16±2.59^c^	57.43±4.66^b^	2.39±0.24^b^,^c^	0.32±0.03^b^	0.74±0.05^b^	109.26^b^
12	15.89±1.44^a^	2.51±0.17^a^	24.33±2.31^a^,^b^	58.04±5.27^a^	2.58±0.26^a^	0.41±0.04^a^	0.93±0.08^a^	111.69^a^
15	14.78±1.39^c^	2.22±0.21^b^,^c^	24.03±2.48^d^	57.49±5.06^b^	2.58±0.24^a^	0.28±0.03^b^	0.66±0.07^b^	109.14^b^
20	15.37±1.52^b^	2.32±0.22^b^	24.58±2.34^a^	56.42±4.77^c^	2.43±0.21^b^	0.02±0.01^c^	0.15±0.01^c^	107.56^c^
25	15.28±1.48^b^	2.03±0.19^d^	24.22±2.26^b^,^c^	57.63±5.13^b^	2.34±0.16^c^	0.00	0.06±0.01^c^	107.21^c^

DDGS, distiller’s dried grains with solubles; PUFA, polyunsaturated fatty acids.

Mean SD, *n*=3. Means in the same column that do not share alphabetic superscript show significant difference at 0.05 level according to Duncan’s multiple range test.

a–d Means in the same column per parameter with different letters are significantly different at *P*<0.05 by Tukey’s range test.

**Table 4:** Effect of incubation time on PUFA from *M. alpina* for SSF on DDGS at 12°C

	PUFA (mg/g dry substrate)							
	
Time (days)	16:0	18:0	18:1	18:2	18:3	20:5	22:6	Total
2	14.87±1.25^d^	2.04±0.22^c^	25.67±2.47^a^,^b^	56.88±5.28^b^	2.16±0.19^b^	0.09±0.01^c^	0.32±0.03^d^	107.42^c^
3	14.99±1.39^d^	2.26±0.23^a^,^b^	25.59±2.27^b^	57.19±5.09^a^	2.13±0.24^b^	0.14±0.01^c^	0.51±0.03^c^	108.29^c^
4	15.72±1.51^b^	2.01±0.25^c^	25.48±2.26^b^	56.07±4.97^c^	1.96±0.16^c^	0.27±0.01	0.76±0.04^b^	110.58^b^
5	16.02±1.47^a^	2.28±0.26^a^,^b^	25.74±2.31^a^	56.18±5.17^c^	2.49±0.22^a^	0.45±0.02^a^	0.92±0.05^a^	111.68^a^
6	15.87±1.45^b^	2.31±0.18^a^	24.93±2.29^c^	57.04±4.83^a^,^b^	2.36±0.20^a^,^b^	0.41±0.02^a^,^b^	0.87±0.06^a^,^b^	110.86^b^
7	15.26±1.43 c	2.26±0.21^a^,^b^	24.27±2.34^d^	56.13±5.28^c^	2.05±0.17^c^	0.31±0.02^b^	0.65±0.05^b^,^c^	108.43^c^

DDGS, distiller’s dried grains with solubles; PUFA, polyunsaturated fatty acids.

Mean SD, *n*=3. Means in the same column that do not share alphabetic superscript show significant difference at 0.05 level according to Duncan’s multiple range test.

a–d Means in the same column per parameter with different letters are significantly different at *P*<0.05 by Tukey’s range test.

### Effect of initial substrate moisture content on PUFA content

**Fig. 1:**
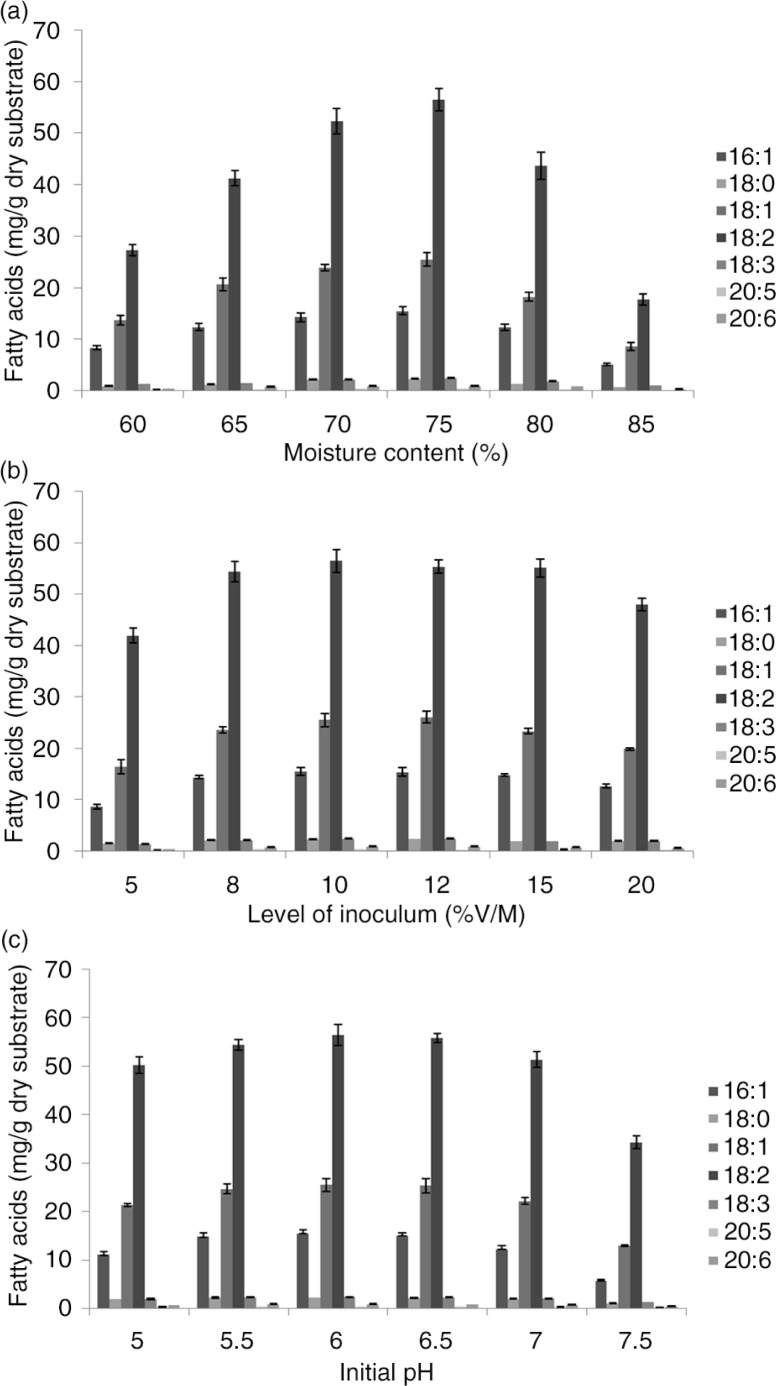
Effects of initial moisture content, inoculums concentration, and initial pH on PUFA production from *Mortierella alpina* by SSF using DDGS as the substrate after 10-day incubation.

A high PUFA yield was attained with an initial moisture level of 75% (Fig. 1a). The moisture content of the SSF substrate affects dissolved oxygen and, consequently, the biosynthesis and secretion of products (36). Low moisture content decreases the solubility of nutrients in the substrate. Also, low degree of swelling and low water activity limit the growth of microorganisms (37). This explains low PUFA content at low moisture content (Fig. 1a). An increase in moisture level is believed to reduce substrate porosity, thus limiting oxygen transfer (38). Therefore, the moisture content of the medium should be controlled within a suitable range. The most suitable moisture content of the medium for PUFA production of *M. alpina* was 75%.

### Effect of inoculation volume on DDGS SSF and PUFA content

The inoculation volume significantly affected fermentation, considering the maximum PUFA production was obtained at 10% (v/w) inoculum compared with other higher inoculation volumes. The results are presented in Fig. 1b. A lower inoculum level may give insufficient biomass, causing reduced product formation, whereas a higher inoculum level may produce too much biomass, leading to poor product formation.

### Effect of initial pH on DDGS SSF and PUFA content

The pH level is another important factor affecting the growth and product yield during SSF. Each microorganism has an optimal pH and pH range for its growth and activity. The initial pH values were adjusted with the addition of HCl or NaOH to 5.0, 5.5, 6.0, 6.5, 7.0, or 7.5 to evaluate the effects of the initial pH value of the solid substrate on PUFA synthesis. Microbial growth achieved a high rate in the pH range of 5–7.5 and decreased dramatically when the pH falls out of this range. Maximum PUFA production was obtained at pH 6.0 (Fig. 1c).

### Effect of additional carbon source on DDGS SSF and PUFA content

**Fig. 2:**
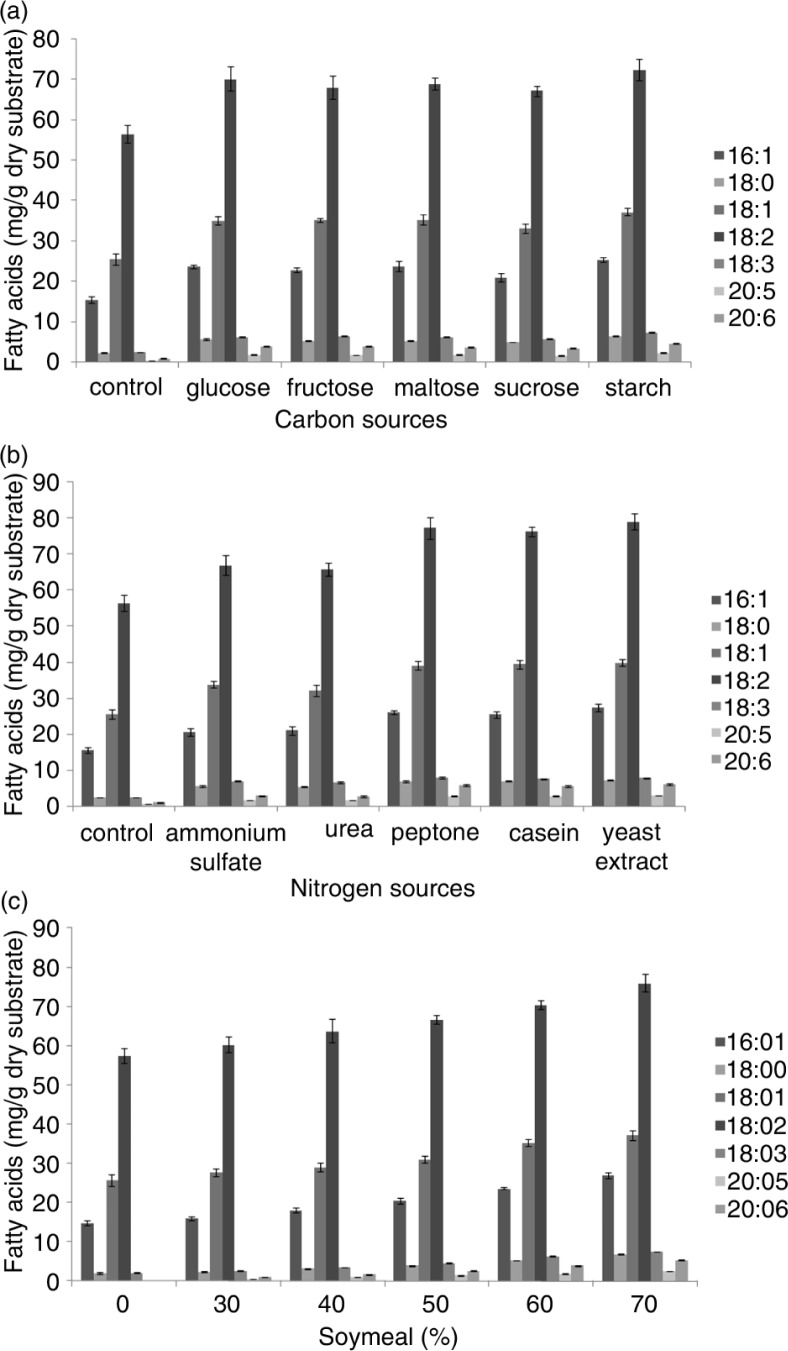
Effects of carbon sources addition, nitrogen sources addition, and soybean meal addition in DDGS on PUFA production from *Mortierella alpina* by SSF after 10-day incubation.

The substrate moisture content was maintained at 75%, the pH at 6.0, and the inoculation volume at 10% (v/w). All samples were initially incubated for 5 days at 20°C and then at 12°C for an additional 5 days. PUFA production was assayed for the control with DDGS alone as the substrate. Very low PUFA were obtained. The TFA obtained were about 103.52 mg/g dry substrate, composing of 0.42 mg EPA and 0.95 mg DHA, during the 10-day incubation. The addition of different sugars (1% w/w) into DDGS resulted in better PUFA production, the highest was obtained with starch (Fig. 2a), followed by glucose, maltose, fructose, and sucrose. With starch, TFA obtained were about 164.25 mg/g dry substrate, composing of 2.34 mg EPA, 72.38 mg LA, 7.48 mg ALA, and 4.66 mg DHA, during the 10-day incubation.

### Effect of additional nitrogen source on DDGS SSF and PUFA content

The nitrogen source or nitrogen availability is an important factor in microbial growth and enzyme production. Since DDGS led to poor fungal growth, different nitrogen sources were added into DDGS to further optimize SSF efficiency. Figure 2b shows the differences in PUFA contents produced with various nitrogen sources. The results showed that PUFA production in nitrogen-supplemented DDGS increased remarkably in comparison with the control. Among these nitrogen sources, the addition of yeast extract yielded the highest PUFA, about 182.34 mg/g dry substrate including 2.84 mg EPA, 79.03 mg LA, 7.78 mg ALA, and 6.05 mg DHA, after the 10-day incubation. Peptone yielded the second highest PUFA yield, followed by casein. Furthermore, organic nitrogen sources were significantly better than inorganic nitrogen sources, indicating that organic nitrogen sources may be more suitable for the growth of *M. alpina* and PUFA formation.

### Effect of soybean meal addition in DDGS on PUFA content

PUFA production was relatively low in DDGS with poor growth. The strains grew better when soybean meal was added to DDGS, as mentioned early. PUFA production also increased with increasing soybean meal content. PUFA production from fermented DDGS with different soybean meal concentrations is presented in Fig. 2c. A positive relation between PUFA and soybean meal percentage in DDGS was observed. The addition of 70% soybean meal to the DDGS substrate yielded 175.16 mg of TFA, including 2.49 mg EPA, 75.94 mg LA, 7.52 mg ALA, and 5.26 mg DHA for 10-day incubation.

### PUFA enrichment and proportion in chicken breasts and liver

The changes in the enrichment and proportion of PUFA in chicken breasts and liver are shown in Table 5. In the test groups fed with SDDGS and vegetable oil-added ration, the PUFA contents in the chicken breasts and liver were higher than those in the control group (*p*<0.05). Table 5 shows that the proportions of ω-6 and ω-3 in chicken breasts in Test Groups 1, 2, 3, and 4 were 36.98, 31.51, 18.15, and 12.63% lower than that of the control group, respectively. The content of ω-6 in Test Group 3 was significantly different from that of the control group (*p*<0.01). In addition, the proportions of both in liver and in the test groups were slightly higher than that of the control group, with the values for Test Groups 1, 2, and 3 higher by 0.76, 0.50, and 0.31%, respectively. A significant difference in the ω-6 content in the liver was observed among the test groups (*p*<0.05). No difference was found in the ω-6 content in chicken breasts among Test Groups 1, 2, and 4. The ω-3 content in the liver of Test Group 1 was significantly different from that of the control group (*p*<0.01). Furthermore, the ω-3 content was significantly different among the test groups (*p*<0.05).

**Table 5:** PUFA enrichment and proportion in chicken breasts and liver

	Liver			Chicken breasts		
		
Group	ω-6 (g/100g TFA)	ω-3 (g/100g TFA)	Proportion	ω-6 (g/100g TFA)	ω-3 (g/100g TFA)	Proportion
1 (SDDGS)	24.50±2.62^a^	4.01±0.64^a^	6.11:1^a^	23.70±3.14^a^	0.94±0.33^a^	25.21:1^a^
2 (3% rapeseed oil)	22.70±2.87^b^	3.88±0.71^b^,^c^	5.85:1^b^	22.40±2.72^c^	0.63±0.45^b^	30.68:1^b^
3 (% soybean oil)	22.30±1.93^b^	3.94±0.43^b^	5.66:1^c^	22.90±2.44^b^	0.52±0.17^c^	44.04:1^c^
4 (3% peanut oil)	21.20±2.46^c^	3.98±0.35^a^,^b^	5.33:1	22.80±2.91^b^,^c^	0.46±0.25^c^	49.56:1^d^
Control group	17.40±2.22	3.25±0.62	5.35:1	19.90±2.78	0.32±0.28	62.19:1

PUFA, polyunsaturated fatty acids; SDDGS, soybean distiller’s dried grains with solubles; TFA, total fatty acids.

Mean SD, *n*=3. Means in the same column that do not share alphabetic superscript show significant difference at 0.05 level according to Duncan’s multiple range test.

a–d Means in the same column per parameter with different letters are significantly different at *P*<0.05 by Tukey’s range test.

## Discussion

Nitrogen source supplement could provide additional nutrients for microbial growth and consequently increase PUFA production. Ben-Amotz (39) indicated that high concentrations of nitrogen source could stimulate EPA production. Nitrogen content of the solid substrate could stimulate ω-3 PUFA production, especially EPA. The organic nitrogen sources and inorganic nitrogen sources were used to improve PUFA production by SSF using DDGS as the substrate. Some researchers have suggested that different strains have different preferences for either inorganic or organic nitrogen for growth and PUFA production, although organic nitrogen sources are usually used for PUFA production (40). In our studies, PUFA production was higher when organic nitrogen sources were used (Fig. 2b and c). Furthermore, organic nitrogen sources were proven more effective than inorganic nitrogen sources in enhancing cell growth and PUFA production. Thus, the soybean meal and DDGS mixture showed good potential as base substrate for PUFA production with *M. alpina*. Sajbidor et al. (41) and Jang et al. (42) reported that carbon/nitrogen ratio of solid substrate was very important in PUFA production.

PUFA production of the fungi was significantly influenced by the moisture content of substrate in SSF. The moisture content in SSF substrate affects dissolved oxygen and thus the biosynthesis and secretion of PUFA. Low moisture content causes a reduction in the solubility of nutrients in the substrate and a low degree of swelling (37), and low water activity limits the growth of microorganisms (43). Lekha and Lonsane (44) also indicated that high moisture contents decreased the porosity and the gas exchange, induced the loss of particle structure and the production of stickiness, reduced the gas volume, and enhanced the aerial mycelium formation. Jang et al. (45) reported that the initial moisture content of solid substrate ranging from 60 to 65% was good for PUFAs production. Moisture content at 70–75% favored the production of ω-6 series PUFAs; while moisture content at 60–65% was good for the production of ω-3 series PUFA. Therefore, we determined that the moisture content of 75% is most suitable for PUFA production in our system (Fig. 1).

The suitable range of ratios between ω-6 and ω-3 recommended by WHO are 1:1–8:1 (46), that by USA are 4:1–15:1 (47), respectively. However, with more demand for ω-3 due to health concerns, this ratio needs to be further reduced (48, 49). This study shows that the proportion of both in the liver was within the suitable range. The proportion of these two fatty acids in chicken breasts was obviously beyond the suitable range. The result indicates that, after adding either of the three vegetable oils and SDDGS, the increment in ω-6 content was higher than that of ω-3. This observation may be related to the different LA contents in the three vegetable oils and SDDGS. These fatty acids, after being absorbed through the intestines of domestic birds, are transported to the liver, and go into the tissue. During this period, palmitic acid, saturated fatty acids can be converted into linoleic and linolenic acids through desaturase and chain extension, while stearic acid can be converted into oleic and LAs. Therefore, after adding vegetable oil and SDDGS into the feed, the proportions of ω-6 and ω-3 in the liver tended to stabilize. Furthermore, the proportions of ω-6 and ω-3 in chicken breasts fed by SDDGS were more close to the suitable range, indicating that SDDGS as a feed for chicken breasts is more helpful in balancing ω-6 and ω-3 in chicken breasts than vegetable oil.

The balance between ω-6 and ω-3, which is very important for stable internal environment and normal growth, can reduce the incidence of cardiovascular diseases and other chronic diseases, and is conducive to mental health (50–54). Both of them cannot be converted in but can interact with the body (55–57). Therefore, the focus should be given on their proportion while their adequate supply is ensured. Many experiments have verified that regulated feed was feasible to produce poultry products with high ω-3 and controlled ratio of ω-6 and ω-3 (58). These studies provided a reliable basis for further investigation and production of poultry products rich in ω-3.

## Conclusions

In this study, soybean meal and DDGS were successfully utilized as SSF substrates for PUFA production by *M. alpina*. Higher biomass and PUFA yield were achieved using DDGS supplemented with soybean meal compared with DDGS alone. PUFA production can also be optimized by added carbon and nitrogen sources, especially organic nitrogen, for the growth of *M. alpina* and PUFA production. SSF enrichment of PUFA is feasible using *M. alpina* as the starting strain. The product from this SSF process improved the laying rate, increased the PUFA contents in chicken breasts and liver, and enhanced the proportions of ω-6 and ω-3. The optimized SSF process we verified is a unique process to maximize PUFA yield by *M. alpina*.
